# Looking Back, Looking Forward at Halogen Bonding in Drug Discovery

**DOI:** 10.3390/molecules22091397

**Published:** 2017-08-24

**Authors:** Lois Mendez, Gabriela Henriquez, Suman Sirimulla, Mahesh Narayan

**Affiliations:** 1 Department of Chemistry, The University of Texas at El Paso, El Paso, TX 79968, USA; lmendez9@miners.utep.edu; 2 Environmental Science and Engineering Program, The University of Texas at El Paso, El Paso, TX 79968, USA; ghenriquez@miners.utep.edu; 3 School of Pharmacy, The University of Texas at El Paso, El Paso, TX 79968, USA; ssirimulla@utep.edu

**Keywords:** halogen bonding, sigma-hole, Lewis base, Lewis acid, drug discovery

## Abstract

Halogen bonding has emerged at the forefront of advances in improving ligand: receptor interactions. In particular the newfound ability of this extant non-covalent-bonding phenomena has revolutionized computational approaches to drug discovery while simultaneously reenergizing synthetic approaches to the field. Here we survey, via examples of classical applications involving halogen atoms in pharmaceutical compounds and their biological hosts, the unique advantages that halogen atoms offer as both Lewis acids and Lewis bases.

## 1. Introduction to Halogen Bonding

Drug discovery has become increasingly computation-intensive. There is a two-fold reason for this. Historic techniques have relied on the identification of principals from traditional remedies, from soil or from bacteria, serendipitously, via optimization of high throughput screens, and by natural product tuning/medicinal chemistry approaches. Hits from these mechanisms have reached a plateau, akin to “Moore’s limit”. By contrast, the “rediscovery” of novel chemistries coupled with the advent of more robust computational techniques to assess the energetics of host:guest interactions have induced a much-needed renaissance in the field.

One such novel bonding chemistry, viz. halogen bonding, has not only permitted ligand-receptor interactions to be further refined and optimized, but also enabled directed design efforts via precise selection of atoms to be incorporated in to the bio-active ligand moieties [1,2,3,4,5,6,7,8,9,10,11,12,13,14,15]. Halogen bonding is commonly termed as X-bonding given its parallels with H-bonding [3]. X-bonds have provided new relevance to the role of halogens in pharmaceuticals beyond the usual metrics (such as enhanced membrane permeability and reduced elimination) that were though to solely improve “druggability” [16,17,18,19,20,21,22,23,24,25]. X-bonding is now understood as a phenomenon in its own right and one that is capable of making precise interactions independent of its role as a hydrogen bonding partner [10]. Importantly, the recent incorporation of halogen bonding into docking algorithms and energetics calculations will undoubtedly pave the way for improved therapeutic outcomes [26,27,28,29,30,31,32,33,34,35,36,37]. Halogen bonding exemplifies the “turning of tables” in drug discovery, wherein analysis, understanding and refinement in silico have driven synthetic approaches to the genesis of 2nd and 3rd generation pharmacophore progeny [16,17,18,19,20,22,23,24,25,38,39]. Halogen bonding, and its applications in ligand-receptor optimization and drug discovery, are discussed in the following sections.

### Origin of Halogen Bonding

**Figure 1:**
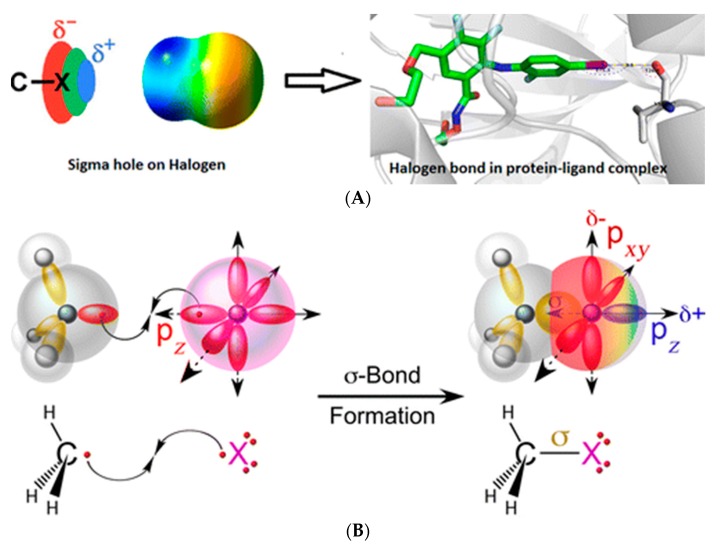
(**A**) The appearance of a sigma-hole (δ+) that can interact with a region of electron density residing in a different atom. Here, the halogen atom (or R-X) is an “electron-poor” species and acts a halogen-bond donor (D). The acceptor (Y) possesses a rich electron density and acts as a nucleophile, thereby completing the halogen bond between the Donor sigma-hole and the acceptor lone pair. Such an interaction is observed in many drug:receptor systems; (**B**) Another view detailing the formation of the σ-hole: The covalent bond (C-X) between the Carbon atom and the halogen (σ-bond, yellow) pairs an electron from the carbon with one from the valence *p_z_*-orbital of the halogen. The electron withdrawing nature of the R-C moiety results in the depopulation of the *p_z_*-orbital of the halogen atom. As a result, it creates an electropositive crown (blue) and flattening of the atomic radius opposite the σ-bond, while the *p_x,y_*-orbitals remain fully occupied, resulting in an electronegative annulus perpendicular to the covalent bond. (**A**) has been reproduced with permission from reference [39] and (**B**) from reference [15].

Consider a bond of the type R-C-X (alternatively, R-X) where C is carbon and X is a halogen (Figure 1A). Note that fluorines rarely participate in X-bonds so X usually refers to the remaining halogens (Cl, Br and I). R is a moiety that is electron withdrawing in nature (for example, an aromatic group). Then, the shared electron pair (bonding pair) between C and X is drawn towards the carbon atom. In other words, the distal region of the so-called Pz lobes of the halo-atom is electron-density depopulated (Figure 1B). The resulting anisotropic distribution of electronic charge (density) around the halo atom is reflected in the formation of a relatively positively charged region called “sigma hole”. The presence of the electro positive sigma hole makes it possible for the halo atom to interact with lone pair electrons of other electronegative atoms in the vicinity. For example, the halogen can non-covalently bond with atoms such as oxygen, nitrogen, sulfur or simply other halogen atoms. Such an interaction constitutes the halogen (X-) bond [4,5,6,7].

An alternative model, termed a “lump-hole” model attempts to remedy inadequacies observed in the σ-hole concept [7,8]. A lump-hole is a proposed local charge depletion at donor terminus of the halogen bond and it can then “halogen bond” with the electronic density of the acceptor. In this review, we will limit ourselves to the sigma-hole description used to explain the origin of halogen bonding.

It is notable that the “quality” of the sigma hole can be further modulated by choice of halo-atom [7]. The largest electropositive density is observed with iodine. The iodinic sigma-hole can be additionally amplified by introducing electronegative (electron withdrawing) moieties at the proximal ends around the C-X bond [7]. For example, a highly fluorinated aromatic ring (R), would influence the already electropositive X-end of the R-X bond. These are some of the “first principles” governing rational drug design. Albeit rudimentary, these building blocks, coupled with an understanding of the thermodynamics and sterics of such interactions, the synthetic limitations and tractability imposed by substitutions, and experimental verification are key tenets that improve “druggability” and thereby enhance clinical outcomes [26,27,28,29,30,31,32,33,34,35,36,37].

## 2. Halogen Bonding in Biological Systems

Halogen bonding interactions in biological systems have attracted widespread interest [11,12,13,14,15,16,17,18,19,20,21,22,23,24,25,26,27]. They have garnered this interest not only from the perspective of basic research but also in clinical endpoints through pharmaceutical design and development. A fundamental understanding of halogen bonding interactions in biological systems involves analysis, both experimentally and theoretically, of bond angles between the donor atom, the halogen, and the acceptor atom and the energetics of such interactions. A comparison of similar properties with H-bonding interactions is particularly useful to gather information about the utility of introducing halogen bonding in lieu of existing hydrogen bonding or in addition to H-bonding. Furthermore, similar to the scenario in small molecules, biological systems are also sensitive to entropic perturbations. In both systems, entropic and enthalpic interactions compete with one another. Thus, the presence of a halogen bond, introduced at the expense of a hydrogen bond, must engage with sufficient enthalpic energy to overcome entropy. The next sections summarize the role of halogen bonding with respect to receptor-ligand interactions within a primarily biological context. Particularly, we will elaborate on those compounds that are of pharmaceutical interest; an area in which a large number of papers have emerged exploiting halogen bonding interactions between ligands and their receptors [26,27,28,29,30,31,32,33,34]. Of value, in many instances the interactions have been described both qualitatively and quantitatively [12]. The quantitative aspects, which are crucial to engineering binding and enhance drug efficacies, have spurred various research groups to described energy functions and docking algorithms of halogen bonds. However, these remain outside the scope of this review.

### 2.1. Halogen Bonding between Small Molecules and Proteins

**Figure 2:**
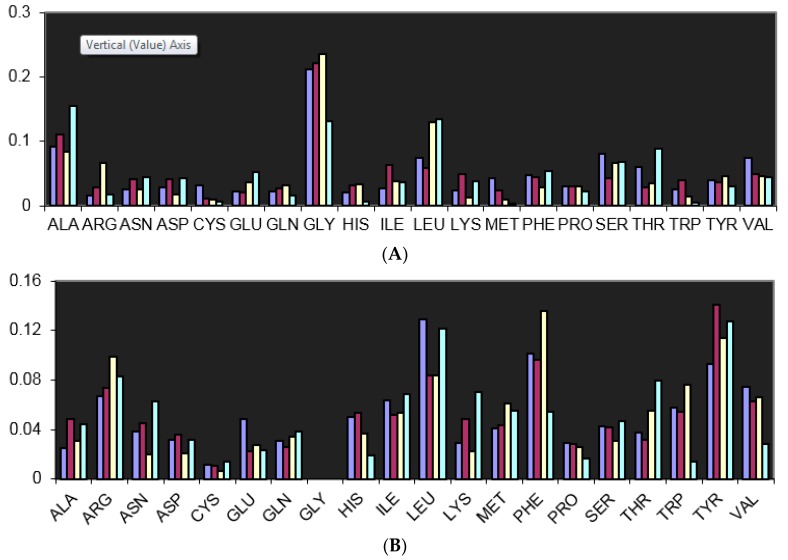
(**A**) Frequency of interactions of halogenated ligands with proteins. The interactions are defined using van der Waals radii between halogen of the ligand and backbone carbonyl or backbone nitrogen of the protein as Lewis bases. The interaction is labeled as being as above at the N-Cα-C of the side chain that is pendant off of the backbone.The data is normalized to the maximum number of interactions made by each halogenated ligand; (**B**) Frequency of interactions of halogenated ligands with proteins. The interactions are defined using van der Waals radii between halogen of the ligand and side chain Lewis bases (oxygen, nitrogen, and sulfur). Also included are interactions as above with the π electron rich aromatics residues (Phe, Tyr, Trp and His). The data is normalized to the maximum number of interactions made by each halogenated ligand (the figure has been reproduced with permission from [39]).

The docking of halogen containing pharmaceutical compounds to their biological targets have been analyzed by a number of groups [26,27,28,29,30,31,32,33,34]. As a starting point, halogen bonding interactions between halogens in drugs and the backbone and side-chain donors of amino acids have been mined from the PDB [38,39]. These studies have primarily relied on strictly restricting “halogen bonds” to the sum of the Van der Waal’s distances between the participating atoms. Among the structures deposited in the PDB, halogen atoms in pharmaceutical compounds were found to interact most frequently with the backbone Lewis bases, viz. O and N, of glycine (Figure 2A).

This is an expected outcome given the absence of a side-chain for this amino acid, permitting access to lone pairs of both Lewis bases from either direction. Two independent studies found that the frequency with which the backbone amino and carbonyls of Leu as acceptors for halogen bonding interactions was large despite anticipated steric hindrance from its bulky side-chain [38,39]. The high fraction of observed interactions relative to amino acids with smaller side-chains is likely the result of secondary effects. For example, the hydrophobic interactions between the side-chain of Leu and the hydrophobic moieties in the organopharmacophore could bias the halogen bonding tendencies in favor of the Leu backbone. The remainder of the trend that follows in the frequency of X-bonding interactions is that of Ala and other small-side-chain-bearing amino acids with a flat-lining found for interactions involving bulkier and aromatic side-chains.

The trend for side-chain Lewis bases involved in halogen bonding differed from backbone tendencies [38,39] (Figure 2B). Along with arginine, which likely proffers nitrogen for bonding, the aromatic amino acids were frequently found to be within van der Waals’ distance with the halo atoms. It is likely that the aromatic amino acids behave as non-classical Lewis bases via their pi cloud such as evidenced by Tyr. In this case though, the data did not discriminate between X-piTyr and X-O-H Tyr interactions.

The donor-acceptor bond angles (C-X···Lewis Base) in both sets of interactions were found to span a wide range with only a handful of interactions approaching linearity. Two reasons have been postulated to help reconcile the large extent of non-linearity in the spectrum of halogen bond angles that are made. (1) The electron-rich Lewis-base (acceptor) may polarize and thereby modulate the electron distribution deficit (sigma hole) at the distal (halogen ) end of the R-C-X bond [40]; (2) The probability of the acceptor atom aligning itself to make a linear (180°) approach is relatively small compared to other areas of the sigma-hole spread. Thus, even though it’s energetically favorable to align with the “bulls-eye” of the sigma hole “target”, in practice it may not be the case [9]. The latter reason can particularly be influenced by steric factors that can drive the bond angle.

**Figure 3:**
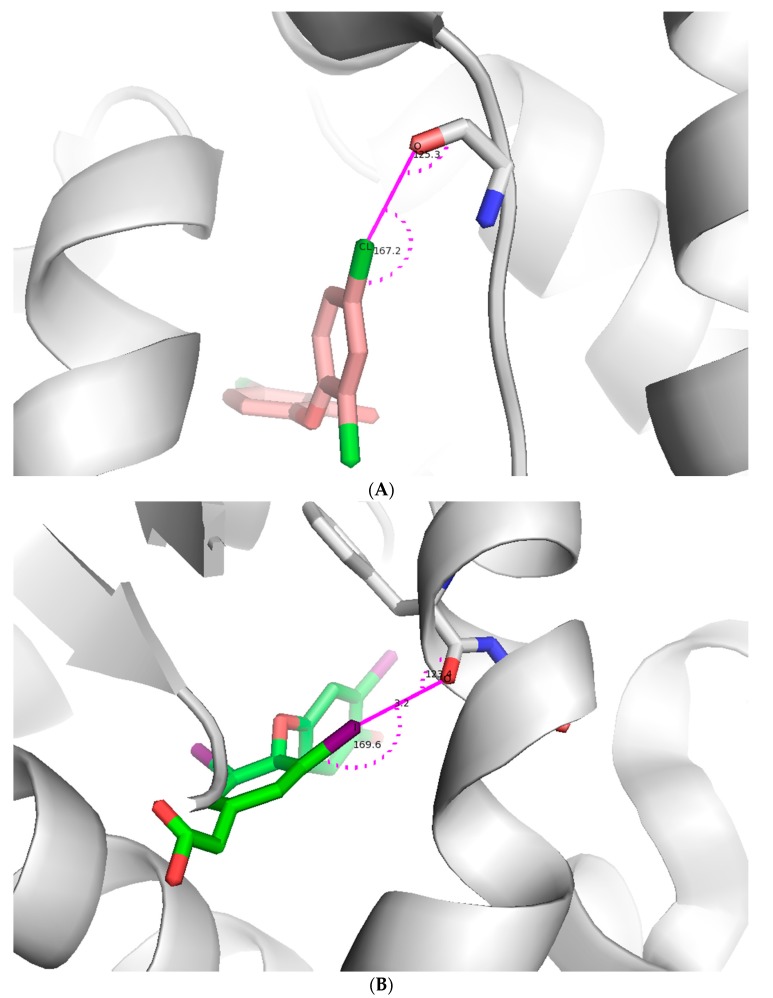

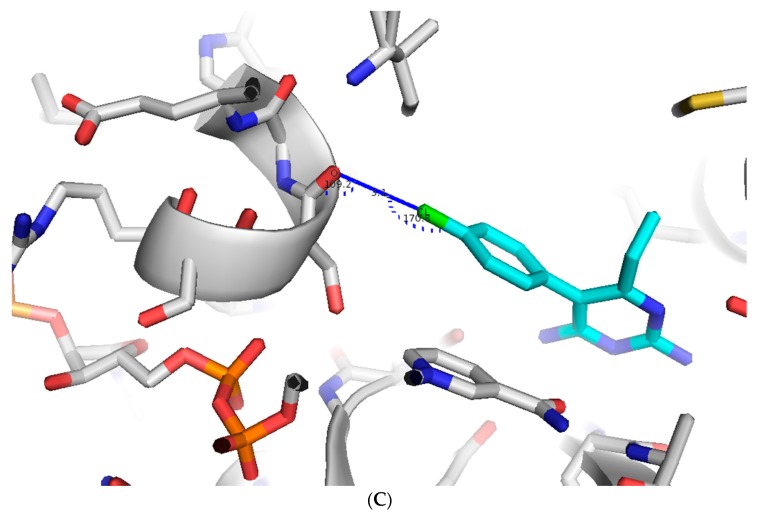
Illustration of a halogen bond in a protein-ligand complex. (**A**) Chlorine forms a halogen bond with main chain oxygen of the protein. The ligand is shown as light pink stick model and protein is shown as both a cartoon model and interacting amino acid as stick model (pdb id: 4ALI); (**B**) Iodine forms a halogen bond with main chain oxygen of the protein. The ligand is shown as an green stick model and protein is shown as both a cartoon and stick model (pdb id: 3JZB); (**C**) Chlorine forms a halogen bond with main chain oxygen of the protein. The ligand is shown as a cyan stick model and protein is shown as both a cartoon and stick model (pdb id: 2B19). Figures were prepared using PYMOL.

Figure 3 shows typical halogen-bonding interactions found in the PDB between halogen-containing pharmaceutical compounds and their receptors. The choice of halogen atom and the donor-acceptor pair can be used to enhance molecular interactions as described in the succeeding sections.

### 2.2. X-Bonds in Drug Discovery: Case Studies

As stated before, much work has been completed in the area of tuning halogen atoms in ligands to avail of better ligand:receptor interactions via enhanced X-bonding. In the following sections we review case studies involving strategies used to improve ligand: receptor interactions by directed design of halogen bonding.

#### 2.2.1. X-Bonding in Cathepsin

**Figure 4:**
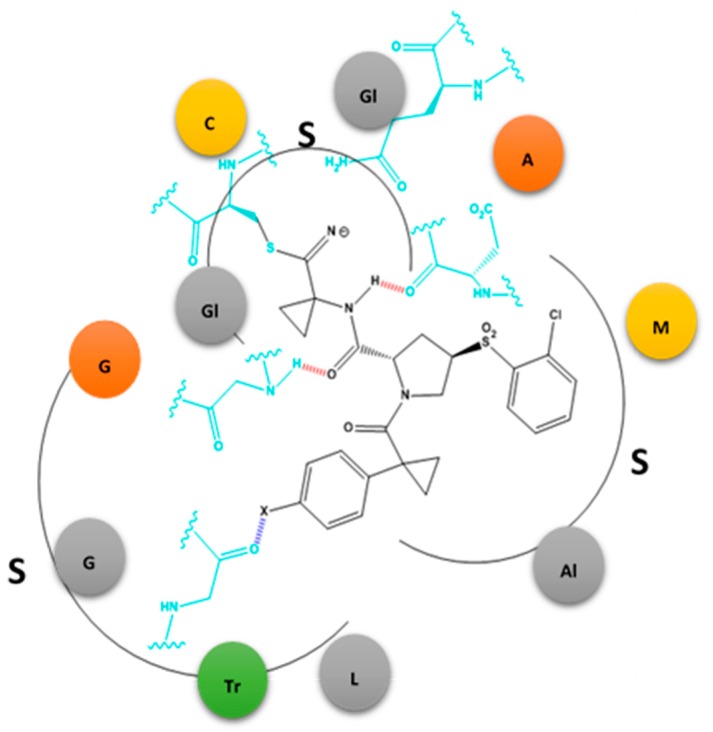
The docking of covalent inhibitors to hCatL (possessing three pockets). The chemical moiety at C4 benzyl is in the 3rd pocket (S3). And approaches the oxygen Lewis base of the carbonyl of Gly 61. If the halogen is Cl, Br, or I, the bond strength increases, resulting in enhanced binding affinity. Figure has been created using data from [15].

A series of halogen-containing inhibitors of cathepsin have been recently formulated, pursuant to the discovery of hydrogen bonding, between select ligands and the active-site of cathepsin [15]. The fine-tuning of the cathepsin binding inhibitors was possible as a result with an outcome that enhanced several-fold the binding constant observed between the starting ligands and their halogenated successors (Figure 4). For example, XB was found to increase in strength with the mass of the halide substituent (Cl < Br < I). By contrast, no favorable bonding was found to exist with organofluorine derivatized compounds. Furthermore, the halogen bonding interaction was found to have stringent geometrical specifications such as a distance between the interacting atoms below the sum of the van der Waals radii and a strong dependence on the O···X-C angle. Nevertheless, in conclusion, the study revealed that establishing a halogen bond enhanced protein–ligand interactions several-fold which translated into a gain in free enthalpy of −∆∆G = 2.6 kcal/mol.

#### 2.2.2. Sildenafil

**Figure 5:**
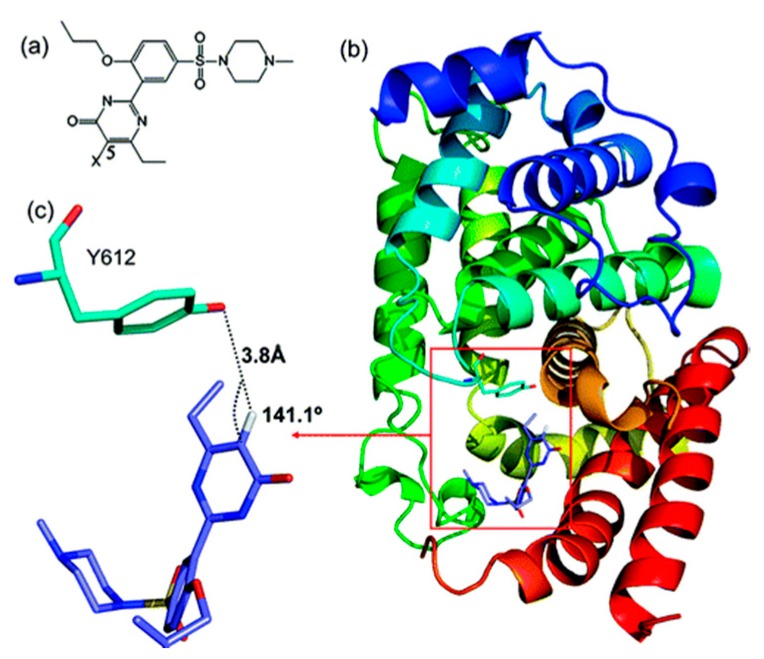
Structure and the binding mode of **1** (X = H) to PDE5 from the docking study. (**a**) Chemical structure of **1**; (**b**) The docked structure of **1**–PDE5 complex. PDE5 is shown in cartoon form with Y612 represented in stick form in the boxed area. **1** is also represented in the boxed area in stick form; (**c**) For clear visualization, the hydrogen bonding between 5C-H in **1** and Y612 in PDE5 extracted from (**b**) is shown (figure has been reproduced with permission from [23]).

Similarly, the first comprehensive thermodynamic and structural characterization of halogen bonding in PDE5−inhibitor interactions has been described exhaustively in terms of their thermodynamic binding parameters (Figure 5) [23]. In this recent “textbook” study, Xu et al investigate the binding of sildenafil with its receptor with the overall aim of designing better binding efficacies between the sildenafil successors and the receptor [23]. Starting with the X-ray structure to examine the atomic details of the contacts between the receptor and the drug, they used a molecular docking program to replace hydrogen atoms in the drug by halogen atoms that could potentially make X-bonds. Using a hybrid approach involving both QM and MM, the authors were able to refine the search to candidates that were subsequently synthesized. The experimental binding energies of the progeny with the receptor were found to be in excellent agreement with the computational predictions demonstrating that it is possible to rationally incorporate halogen bonding to impact drug discovery.

#### 2.2.3. X-Bonding in Unnatural Amino Acid:Protein Complexes

Other groups have studied the influence of halogen bonds in complexes of proteins and halogen-containing non-natural aminoacids. In their study, attention was focused on the interface between proteins and halogen-containing non-natural amino acids [41]. This study revealed that X-bonding can occur by direct amino-acid:protein contact or via a water-mediated mechanism. In the latter mechanism, a water molecule formed a bridge between the amino acid and the polymer, simultaneously engaging in halogen- and hydrogen bonds. The data also indicated that in amino acid–protein complexes the halogen atoms, despite being usually surrounded by hydrophobic residues, could also engage in hydrogen bonding interactions with hydrogen bonding-donors.

The quantification of such bonds in binary complexes, as well as its in silico prediction is particularly useful in advancing our understanding of the mechanism by which the ligand achieves specificity. It also underscores knowledge of the binding pocket which in turn enables selectivity among drug candidates.

#### 2.2.4. The Donepazil Study

It is abundantly clear that halogenation can be used to further efforts in modulating drug design. By virtue of its ability to facilitate transfer of drug moieties across biological barriers (plasma membrane, blood brain barrier, etc.), filling of small hydrophobic pockets in protein topologies, impacting lifetimes and easing drug uptake, halogenated pharmaceuticals have been deemed to be extremely versatile and malleable in their applications. In comparison with other halogenations, the tuning via fluorination and carbon trifluoromethylation have made inroads in medicinal chemistry [42,43,44,45]. Of particular interest is the stabilization of interactions between drug molecules and their protein/DNA targets achieved through relatively improved bonding the donor-acceptor couples through charge distribution and the shuffling of halogens to yield improved binding parameters which has been successfully used in recent studies [46,47,48,49,50,51,52,53,54,55,56,57,58,59].

For example, a series of donepezil drug derivatives designed to inhibit acetyl cholinesterase (AChE) were prepared using the halo atom as a functional “director” [60]. The authors of this study were able to use Density Functional theory (DFT) to optimize the interaction between the parent drug and its halo-modified derivative (both the chair and boat conformers; B3LYP/MidiX and B3LYP/6-311G + (d,p) level of theories). Attributes such as “charge distribution, dipole moment, enthalpy, free energy and molecular orbitals” of the ligands of interest were also investigated to in an attempt to better understand how the halogen-directed modifications impact the ligand structure and impacted the interactions with the receptors [61,62,63,64,65,66,67,68,69]. In addition, molecular docking calculations were performed in the same study to obtain information on similarities and differences between the binding modes of unmodified and halogenated chair-formed ligands [60,67,68,69]. The results revealed that donepezil and its halo-derivatized progeny interacted with hydrophobic gorges and anionic subsites of AChE [61]. The trifluorinated ligand possessed the most desirable free energy. The receptor-ligand interactions were found to be mostly hydrophobic and of the π-stacking type. These studies revealed that fluorine, chlorine and trifluorinated ligands were the most effective and specific among the tested AChE inhibitors, interacting strongly at both the catalytic sites of AChE. This study was then followed by examining the pharmacokinetic parameters of the parent and halo-substituted progeny.

### 2.3. Some Other Aspects and Applications of Halogen Bonding

A theoretical study involving negatively charged donor moieties were found to be fully capable of important X-bonding interactions because in many situations both participants, viz., the organohalogen and nucleophile are both deprotonated (i.e., anionic in nature) and this may result not only in the loss of the σ-hole functionality but also a potential repulsion between the “pairs” [70,71]. The authors noted a remarkably high degree of variability in the distance between the center of the negative charged donor and the halo atom. To further attempt to define a clear relationship among the parameters, a series of model systems comprising 4-halophenyl-conjugated polyene acids and ammonia, were designed to examine the effect, if any, of distance on halogen bonding as a function of solvent chemistry. QM indicated a direct relationship between distance and bond strength. The authors deconvoluted the energetics, in the process revealing that the majority contribution to the binding energy resided in electrostatic interaction, followed by orbital interaction (42–36%). Furthermore, “natural bond orbital calculations showed that electron transfer takes place from the acceptor to the donor, whereas the halogen atom becomes more positive during the bonding, which is in agreement with the result of neutral halogen bonding”. Theoretical work (QM/molecular mechanics) demonstrated that “the polarity of binding pockets makes all of the interactions attractive in a protein system”. This lends “tunability” to the strength of the halogen bonding interaction between the halo atom and the negatively charged donor via variations in the distance between the negative charge center and halogen atom. Further tuning can be achieved through variations in the solvent medium [69,72,73]; though this latter feature may not have limited relevance in a physiological milieu that drugs operate in.

A survey of the entire database clearly indicated that halogen bonding-like interactions between two anions are possible. QM computations involving small model complexes of halobenzoates and propiolate suggested that interactions of the type “anion–anion halogen bonding” are unstable in vacuum [71,72]. By contrast, the same calculations revealed that similar interactions were favorable in solvents. Impressively, the QM optimized X-bonding link between the participating anions is shorter than that in a neutral system, indicating a possibly stronger halogen bonding interaction, as was evidenced by computed binding energies. Energetics’ analysis on multiple ligand-receptor system indicated that anion–anion X-bonding could assist in docking with values approximating 3 kcal/mol making anion–anion X-bonds both a stabilizing factor and of utility to drug design.

#### 2.3.1. Halogen Bonding in Nucleic Acids

Not much work has been done in the area of halogen bonding in nucleic acids. Nevertheless, a recent survey examining the role of halogen bonding in nucleic acids finds that the phosphate backbone oxygen is clearly the most common halogen acceptor [74]. In this study, the authors identified 21 X-bonds among deposited structures with the majority of the intremolecular bonds formed by halogenated nucleobases such as bromouridine with near-ideal geometries. This study could herald a new era in the development of molecules that could chelate DNA and open up additional therapeutic potentials that is enabled by halogen bonding.

#### 2.3.2. Halogen Bonding and Anesthetics Specificity

The efficacy of the anaesthetic, propofol (PFL, 1-hydroxyl-2,6-diisopropylbenzene), was investigated using its halogentaed counterpart, anesthetically inactive fropofol (FFL, 1-fluoro-2,6-diisopropylbenzene), as a contrast [75]. While PFL binds to receptors via hydrogen bonding, FFL is inactive (though it is able to bind via halogen bonding intreactions). The study pointed out that “multiple specific interactions rather than just hydrogen or halogen bonds must be taken into account to explain the different anesthesia endpoints caused by PFL and FFL”. The work highlighted the importance of retaining hydrogen bonds as opposed to simply replacing them with halogen bonds. It showed the significance of taking into account multiple bonding interactions while designing drug-receptor interactions.

#### 2.3.3. Halogen Bonding and Drug Repositioning

Drug repositioning is an outcome of the relatively slow pipeline of new drugs that make it to the market. An interesting study recetly led to the repurposing of rafoxanide and closantel as potent B-Raf V600E inhibitors via molecular docking with improved halogen bonding scoring functions [76]. The experimentally determined IC50 values of 0.07 μM and 1.90 μM, respectively, made it comparable to vemurafenib (IC50: 0.17 μM), a marketed drug targeting B-Raf V600E. These studies vindicated the notion that that a robust halogen bonding scoring function is essential for accurate molecular interaction prediction. The outcome in this case being the repositioning of drugs with heavy halogen atoms in their molecular structures.

#### 2.3.4. Halogen Bonding in Directed Biosynthesis

Halogen bonding use has found application in drug biosynthesis as opposed to its traditional exploitations in enabling drug discovery, binding efficacy, and druggability. In a novel study, authors were able to exploit X-bonding in the nitrilase-catalyzed synthesis of *ortho*-, *meta*-, and *para*-chlorophenylacetic acid [77]. Different distributions of halogen bond induced changes of substrate binding conformation and affected substrate selectivity. The authors report that the engineering of the halogen interaction led to alterations in the substrate selectivity of the enzyme. The implication from this work is that X-bonding can be used to tune biosynthesis and “should be used as an efficient and reliable tool in enzymatic drug synthesis”.

## 3. Discussion

**Figure 6:**
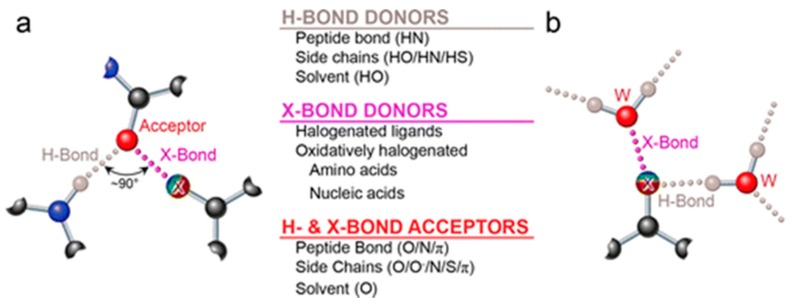
The versatility of halogens and additionally non-halogen Lewis bases in chemical bonding. (**a**) A Lewis-base acceptor (red, Oxygen) can make halogen bonding interactions with the sigma-hole end of a halogen atom (x; bluish to red indicates electrostatic potential trend from +ve to –ve) while simultaneously proffering other lone pairs for H-bonding; (**b**) A halogen atom involved in both X-bonding via its sigma hole and H-bonding via its lone pairs). Both interactions show are with water molecules (figure has been reproduced from [15]).

Clearly there is a lot of excitement in the community about the contribution of halogen bonding to drug discovery. Given that the field is still somewhat in its infancy, the computational approaches to clearly defining the energetics and geometry of X-bonding between a ligand and a receptor are still undergoing development. It is clear from the aforementioned examples that no single technique is satisfactory to clearly pin-point where a halogen must reside and which hydrogen it must replace. As a result there are several “hits and misses” with the nature of the publishing field such that we only see the “hits”. There are several aspects involved in X-bonding that the computational field has yet to reconcile. One example is the replacement of weakly acidic hydrogens with halogens. Weakly acidic hydrogens are those hydrogens that are not involved in traditional hydrogen bonding but may at best associate with Lewis bases via “weak hydrogen bonding interactions”. For example these are hydrogens around conjugated ring structures with a result that the bond is not very polar as would be the case in normally in F, O, N linkages with H. The ability to exploit these weak H-bonds by replacing them with halogen bonds would not comprise the ligand:receptor interaction as would be the case when replacing a hydrogen bond with a halogen. By contrast, replacement of weakly acidic hydrogens in the ligand with halogen atoms that are positioned to halogen bonding with receptor Lewis bases would further stabilize the ligand:receptor interaction. Thus it is important for computational approaches to be able to calculate the energies associated with weak hydrogen bonding interactions between the ligand and receptor and examine whether replacement of such interactions by X-bonds is feasible. A second aspect is the need to exploit the dual Lewis base–Lewis acid tendency that inherently exists in halogens that are engaged in X-bonding for drug design (Figure 6). 

The ability of halogen bonding halogen atoms to engage in hydrogen bonds that are orthogonal is particularly tantalizing for enhanced binding. This feature is unique to halogen bonding and does not pertain to hydrogens involved in hydrogen bonding. For example, a halogen atom can make a σ-hole induced halogen bonding interaction with a Lewis base in a receptor. At the same time, the halogen atom, by virtue of its lone pair can hydrogen bond with an acidic receptor H-atom. There are other combinations of this such as when the receptor Lewis base can both hydrogen bond and halogen bond to ligand acidic hydrogens and ligand halogen σ-hole. Furthermore, relative to receptor nitrogen and sulfur as Lewis bases, oxygen can act as a “multi-Lewis-base” by hosting Lewis acids and/or halogen bonds simultaneously”. To these reviewers’ knowledge, oxygen is not unique in its ability to serve as a “multi-Lewis-base”, relative to N or S. S has exactly the same outer shell electronic configuration as O and, therefore, its non-bonding electrons should have similar properties. N in delocalized systems have both in-plane nonbonding electrons and out-of-plane π-electron systems. The prevalence of O as an acceptor are likely more to do with how prevalent and accessible the atom is in biological molecules. Thus, while parallels can be drawn between halogen bonding and the well-known hydrogen bonding phenomenon, a key distinction between hydrogen bonds and halogen bonds is that the halogen atom can act as both a Lewis base and a Lewis acid because it possesses both a sigma hole and multiple lone pairs of electrons. However, the hydrogen atom in the hydrogen bond can only act as a Lewis acid. Thus halogen bonding is more versatile than hydrogen bonding

As a result, it becomes important for force fields and search algorithms to accurately identify those moieties than can engage in both X- and H-bonding and exploit them in rational drug design. It is clear that much work needs to be done to fully exploit the arsenal of interactions that are possible via logical introductions of σ-holes via halogen atoms. Nevertheless, in conclusion we are confident that that this is a new dawn in the area of computer- aided drug design [78]. The best is ahead of us and computer are bound to reduce costs associated with the bench to clinic process. They will also be instrumental, hopefully, in reducing the number of false candidates, although more accurate predictions are required to get to that stage.

## 4. Conclusions

The examples above are only a few that of the many that illustrate the rich plethora of applications involving the contributions of X-bonding to drug discovery. It is clear from these examples and the rich repository of computational tools that are available to predict and select for the most rewarding interactions that the future of computational drug discovery is bright. The marriage between computational drug discovery and that of halogen bonding in biological systems has provided renewed impetus and hope for the otherwise thought to be “past-prime” area of drug discovery.
